# Effect of Laser Remelting on Microstructure and Wear Resistance of Zr60702 in an Underwater Environment

**DOI:** 10.3390/ma19183835

**Published:** 2026-09-09

**Authors:** Chengyong Ma, Zhen Li, Jianwei Dong, Qiren Zhao, Zhen Luo

**Affiliations:** 1 Central-Ironand-Steel-Research Institute, Beijing 100081, China; 2 School of Materials Science and Engineering, Tianjin University, Tianjin 300350, China; 3 Department of Physics and Astronomy, University College London, Gower Street, London WC1E 6BT, UK

**Keywords:** Zr alloy, underwater, laser remelting, microstructure, wear resistance

## Abstract

Surface remelting is an effective method for modifying surface microstructures and improving the mechanical properties of metallic materials. However, the influence of an underwater environment on the laser remelting behaviour of zirconium alloys remains insufficiently understood. In this study, commercially pure Zr60702 was subjected to laser surface remelting in air and underwater environments under identical processing parameters. The phase constitution, microstructure, microhardness, and wear resistance of the remelted samples were systematically investigated. The maximum remelting depth decreased from 507.08 μm in air to 191.35 μm underwater because of the enhanced heat extraction and reduced effective laser-energy input in the aqueous environment. Both remelted samples were dominated by α-Zr at room temperature, while underwater remelting produced finer acicular α′-Zr and refined Widmanstätten structures. The maximum hardness values of the underwater- and air-remelted samples reached 636.7 and 546.5 HV, respectively, compared with 198.3 HV for the base material. The wear volumes of the base material, air-remelted sample, and underwater-remelted sample were 7.04 × 10^7^, 6.77 × 10^7^, and 5.66 × 10^7^ μm^3^, respectively. The improved wear resistance of the underwater-remelted sample was attributed to its refined microstructure and increased hardness, which suppressed plastic deformation and ploughing during sliding. These results demonstrate that underwater laser remelting is a promising method for improving the surface properties of Zr60702.

## 1. Introduction

Due to its characteristics of small thermal neutron absorption cross-section, corrosion resistance, and high strength, zirconium and its alloys are widely used in the manufacturing of pipelines and pressure vessel equipment in the nuclear industry, ocean, petroleum, metallurgy and other fields [1]. However, due to the poor wear resistance of zirconium alloys [2], wear failure often occurs on their surfaces and safety performance is reduced when they are in service under severe conditions [3], which in turn places higher demands on the wear resistance of zirconium and its alloys [1]. Researchers have attempted a variety of surface modifications to zirconium alloys to obtain optimal surface properties [4,5], treating a Zr-1 alloy by high-current pulsed electron beam in a high-temperature environment, which suppressed the amount of hydrogen infiltrated into the alloy and effectively improved the hardness of the alloy. Slobodyan et al. [6] used pulsed ion beams to modify the surface of a E110 alloy and the results showed that for pre-irradiated and unirradiated samples, no significant differences in the substrate and welded structures occurred. Evsin et al. [7] investigated the moulding characteristics of plasma-induced E110 zirconium alloy surface oxides as well as the hydrogen-resistance properties and demonstrated that the plasma treatment has the effect of preventing hydrogenation of zirconium alloys.

Another application for surface treatment of zirconium alloys is laser surface remelting, which has been used to treat a variety of other metallic materials [8,9,10]. Laser surface remelting uses a high-energy-density laser beam to scan the surface of the alloy to be processed [8]; the surface area of the material is rapidly melted, rapidly cooled, and a modified layer of a certain thickness is formed on the surface of the alloy with a significant change in its organization and structure relative to the raw material [10]. Yao et al. [11] remelted the surface of a Ti-Zr alloy using a CO_2_ laser, and the results showed that the alloy organization in the remelted zone was refined, and the microhardness and modulus of elasticity were significantly improved compared to the substrate. Chai et al. [12] used a pulsed laser to surface treat a two-phase Zr alloy (Zr-2.5Nb), and by characterizing the microstructure at the location of the laser treatment, as well as testing the hardness of the region, the remelted zone consisted entirely of maraging α-plates of a few hundred nanometers in width and a large number of nano-twinned crystals, and the hardness of the surface region near the centre of the laser beam was significantly increased. Proskuryakov et al. [13] analyzed the hardness and structure of zirconium alloys (E110) after surface modification using laser in air and determined that the laser’s influence on the surface of zirconium alloys forms a uniformly structured high-hardness coating.

Laser remelting processes considered in previous studies have focused on remelting in atmospheric environments to improve alloy surface quality and hardness, but further improvements are needed [14,15]. Compared to the atmospheric environment, the laser in the aqueous environment changes the surface temperature and penetration depth, and the refraction and accelerated heat dissipation of the laser beam in water results in a significantly smaller penetration depth and heat-affected zone. In aqueous environments, the faster cooling rate of alloyed materials results in harder surface properties [16]. Luo et al. [17] studied underwater laser welding of pure titanium and compared it with laser welding in an air environment and found that the aqueous environment affects the solid-state phase transition process, accelerates the cooling of the molten pool, and forms fine acicular martensite, which improves the overall microhardness of the weld. Guo et al. [18] investigated the porosity and mechanical properties of underwater welded butt joints of 304 stainless steel under different laser powers, welding speeds and other process parameters, and the results showed that underwater laser welding has precise heat input, low residual stress and high welding stability.

However, underwater laser remelting of industrially pure zirconium (R60702) has not yet been reported to point out in detail the remelting state and mechanical properties under different process parameters. For underwater operation, multiple surface scans of zirconium alloys placed in water were performed, and the effects of laser remelting parameters, such as laser power, remelting speed, and lap rate, on the organization and properties of zirconium alloys were investigated by orthogonal experiments. In addition, the specimens obtained under the optimal underwater remelting parameters were investigated for comparison with laser remelting in air.

## 2. Materials and Methods

**Figure 1:**
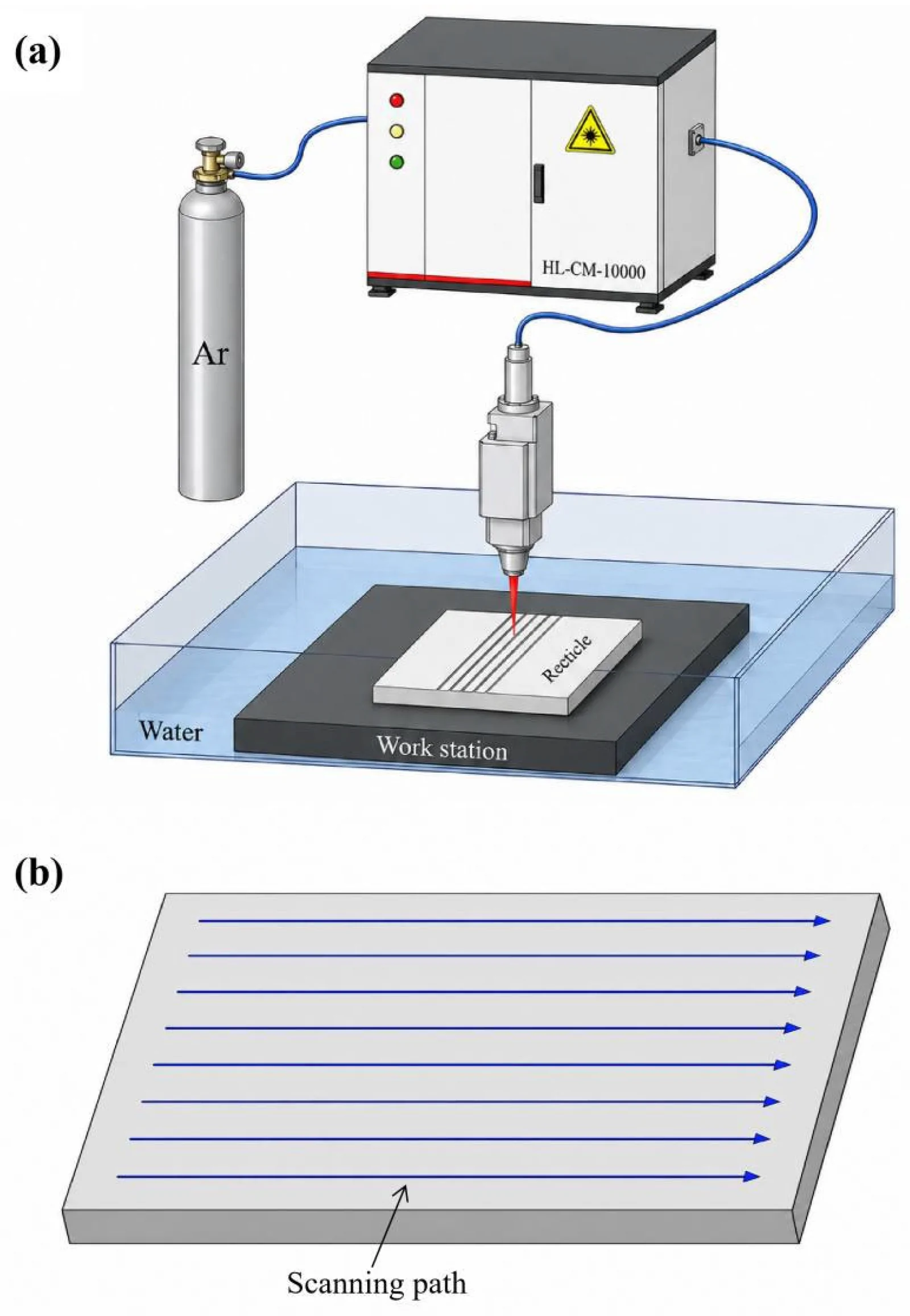
Schematic illustrations of the laser remelting process: (**a**) laser remelting system; (**b**) laser beam scanning pattern.

A commercially pure Zr60702 plate with dimensions of 50 mm × 100 mm × 2 mm was used as the substrate. The chemical composition listed in Table 1 was obtained from the material certificate provided by the supplier rather than independently measured in this study. Laser surface remelting was performed using a HL–CM–10000 continuous-wave fibre laser (Han’s Photonics Technology Co., Ltd., Shenzhen, China) with a central wavelength of 1070 nm. The laser power was 400 W, the beam diameter at the specimen surface was approximately 1.2 mm, the scanning speed was 10 mm/s, and the defocusing distance was +2 mm. Figure 1 shows a schematic of the laser remelting system. Laser remelting experiments were performed on the Zr60702 specimens in air and underwater environments. For underwater laser remelting, the specimen was placed horizontally in the water tank. The vertical distance from the upper surface of the specimen to the water surface was 5 mm, and the initial water temperature was 20 °C. The same water-layer thickness and initial temperature were used for all underwater experiments.

Cross-sectional specimens were removed from the central region of the remelted tracks by wire electrical discharge machining, with the sectioning plane perpendicular to the laser-scanning direction. The specimens were cold-mounted in epoxy resin and sequentially ground using SiC abrasive papers from 80 to 3000 grit. They were subsequently polished using a 1 μm diamond suspension and etched for approximately 10 s with a solution of HF, HNO_3_, and H_2_O at a volume ratio of 2:1:17. After etching, the specimens were rinsed with deionized water and ethanol, dried, and examined by optical microscopy and SEM. XRD measurements were performed using a D8 Advance diffractometer (Bruker, Karlsruhe, Germany) with Cu Kα radiation (λ = 1.5406 Å) operated at 40 kV and 40 mA. The diffraction patterns were collected over a 2θ range of 10–90° with a step size of 0.02° and a scanning rate of 4°/min. The grain morphology and microstructure were observed by optical microscope (OM) and a JSM-7800F scanning electron microscope (SEM) (JEOL Ltd., Akishima, Tokyo, Japan). Microhardness was measured using an HV-1000A Vickers microhardness tester under a load of 200 gf and a dwell time of 15 s. Five indentations were made at representative positions for each condition, with sufficient spacing between adjacent indentations. Wear resistance was determined by MFT-5000 equipment (Rtec Instruments Inc., San Jose, CA, USA). GCr15 was used for the friction pair. The wear tests were conducted under a normal load of 10 N and a sliding speed of 0.5 m/s for 30 min. Each condition was tested independently three times, and the average value was reported. Before and after the wear tests, the specimens were ultrasonically cleaned with ethanol, dried, and weighed using an electronic microbalance (XS105DU, Mettler Toledo, Columbus, OH, USA) with a readability of 0.01 mg. The mass loss was calculated from the difference between the initial and final masses.

## 3. Results and Discussion

### 3.1. Remelting Appearance

**Figure 2:**
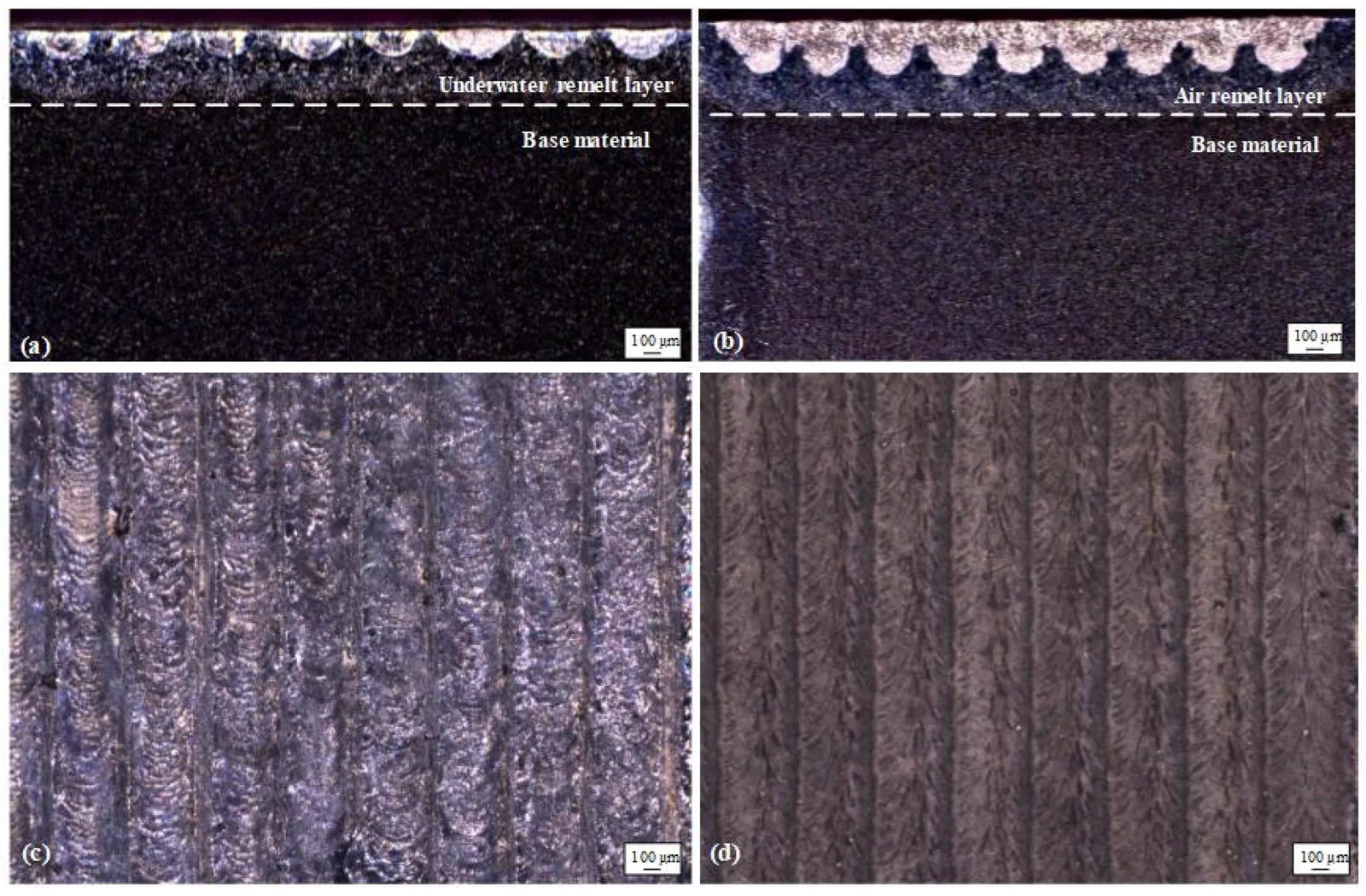
Cross-sectional morphology and surface appearance of Zr60702 after laser remelting in different environments: (**a**) cross-section of the underwater-remelted sample; (**b**) cross-section of the air-remelted sample; (**c**) surface appearance of the underwater-remelted sample; (**d**) surface appearance of the air-remelted sample.

The surface morphology and cross-section of Zr60702 alloy laser remelting under water and in air are shown in Figure 2. The cross-sectional measurements further quantify the effect of the aqueous environment on the melt-pool scale: the maximum remelting depth was 407.08 μm in air, whereas it decreased to only 191.35 μm underwater, indicating that the underwater remelting depth was approximately 47.0% of that obtained in air. Under identical laser power, scanning speed and defocusing distance, this difference indicates that the stronger convective heat transfer and vaporization-induced heat absorption in water substantially shortened the high-temperature dwell time. Meanwhile, laser absorption, scattering and refraction within the water layer, together with perturbations in energy coupling caused by transient bubbles, may reduce the effective energy density reaching the workpiece surface [16,17,18]. These factors collectively limited the downward propagation of the melting boundary, resulting in a shallower remelted layer and a narrower heat-affected zone.

The underwater-remelted surface exhibited a bright appearance and no obvious macroscopic cracks, indicating good continuity of the remelted track under the present processing conditions. This may be attributed to rapid solidification, which shortened the exposure and flow time of the liquid metal, while the confinement effect of water also suppressed excessive spreading of the melt pool. However, the larger temperature gradient may also introduce higher quenching stresses. Therefore, the absence of obvious cracks should be interpreted within the current observation scale. For engineering applications, high-magnification cross-sectional observations, non-destructive testing and residual-stress measurements are still required to exclude the risks of microcracking and delayed cracking.

### 3.2. Microstructure Characteristics

**Figure 3:**
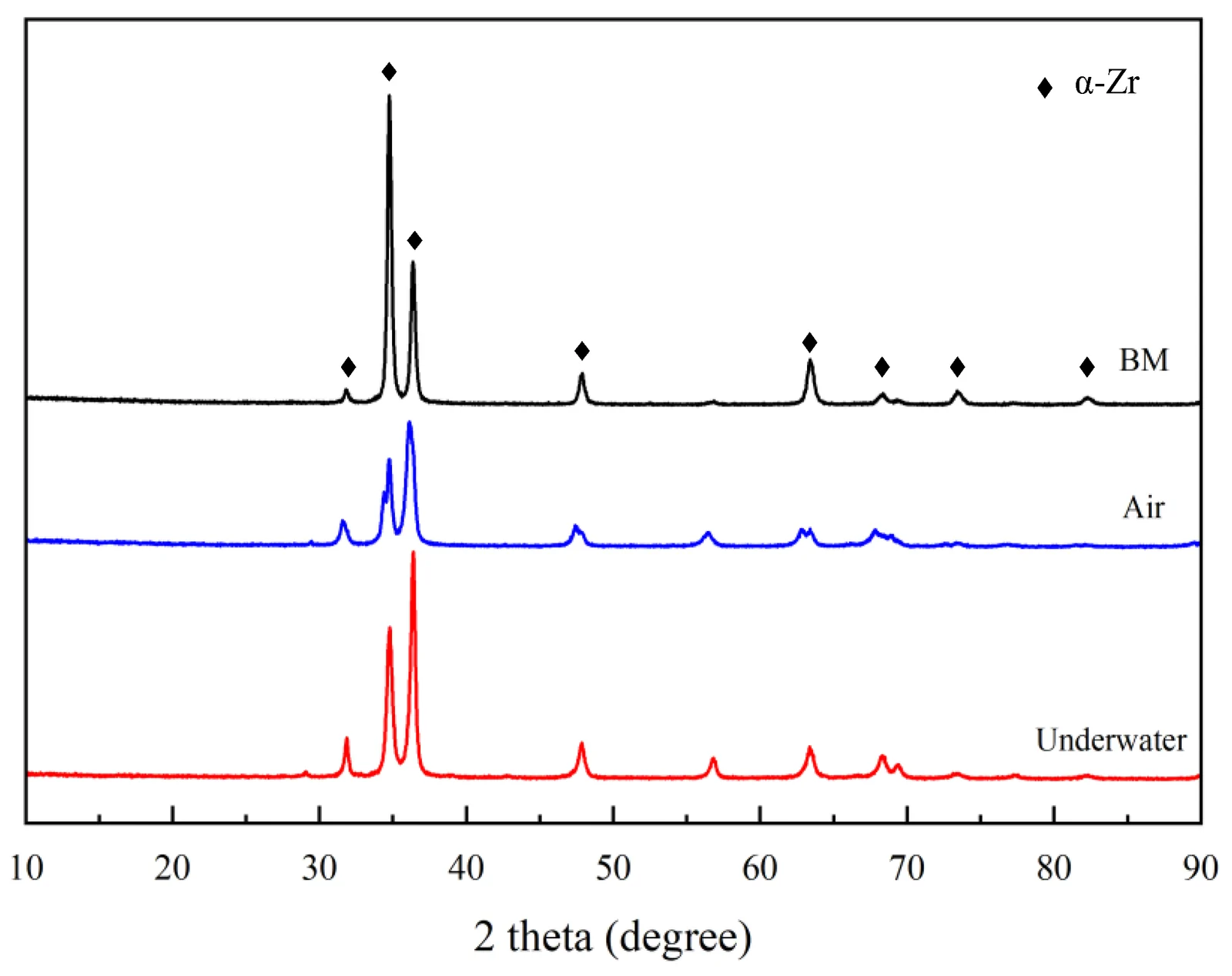
XRD patterns of the base material and the samples remelted in air and underwater environments.

**Figure 4:**
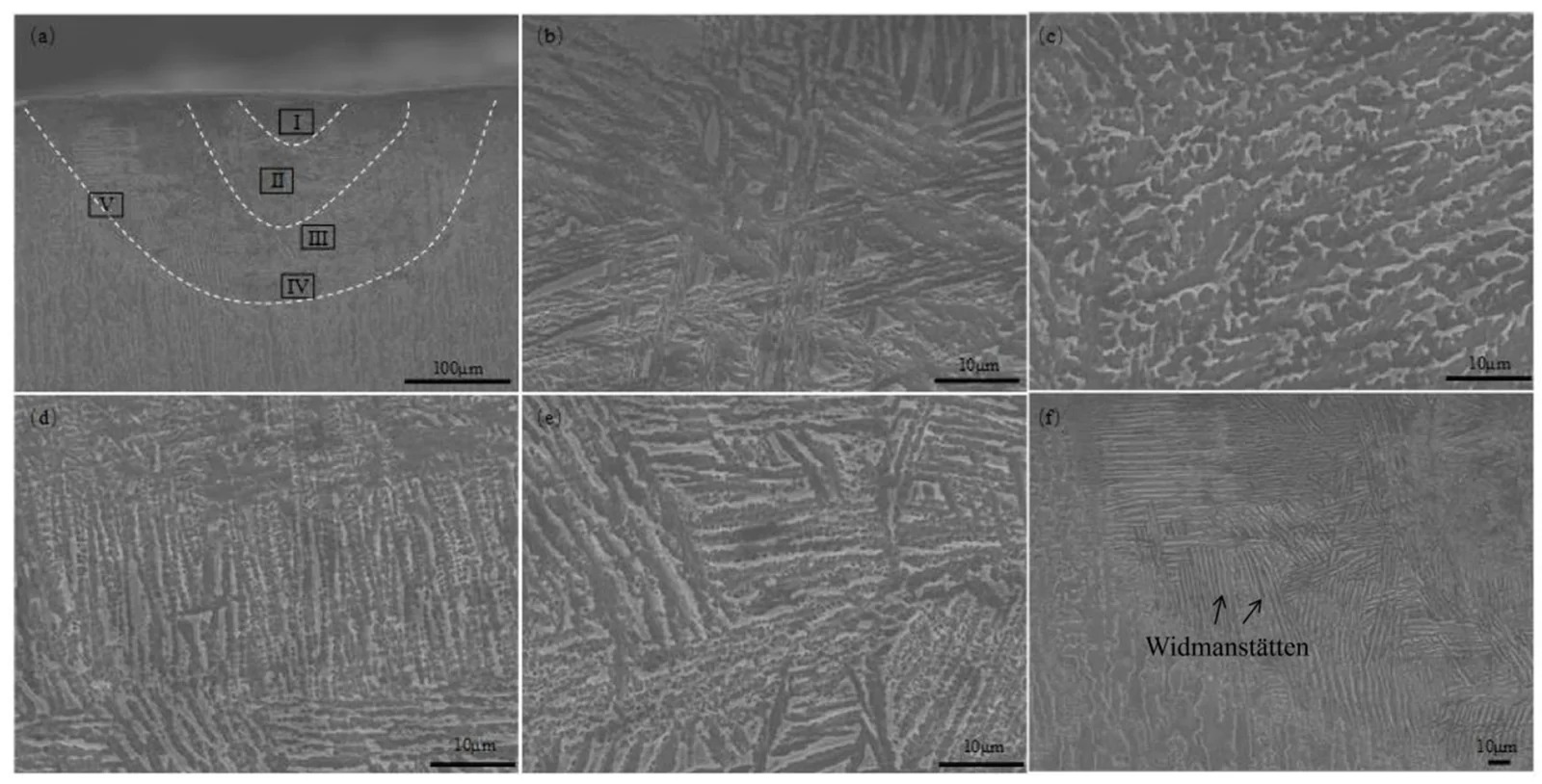
SEM images of the Zr60702 sample remelted in air at 400 W: (**a**) overall cross-section of the laser-remelted zone; (**b**–**f**) enlarged images corresponding to regions I–V marked in (**a**).

**Figure 5:**
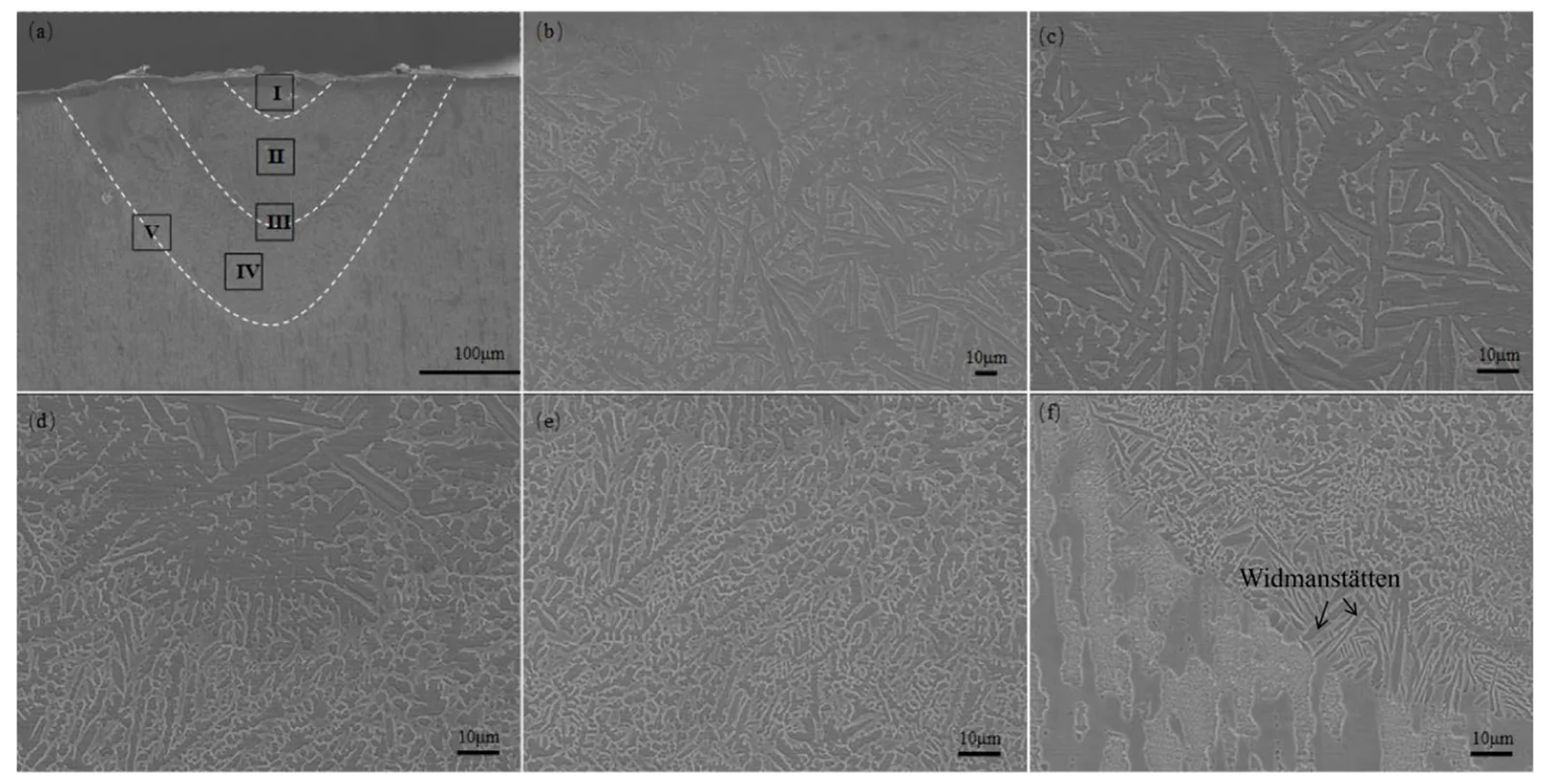
SEM images of the Zr60702 sample remelted underwater at 400 W: (**a**) overall cross-section of the laser-remelted zone; (**b**–**f**) enlarged images corresponding to regions I–V marked in (**a**).

Figure 3 shows the XRD patterns of R60702 processed in air and underwater environments. The dominance of α-Zr diffraction peaks in all three samples at room temperature does not necessarily indicate that no phase transformation occurred during remelting. Upon heating, Zr first transforms from the hexagonal close-packed α phase to the body-centred cubic β phase and then enters the liquid state after exceeding its melting point. During subsequent rapid cooling, the transformation path can be described as liquid → β-Zr → α-Zr/α′-Zr. Because α′ martensite and α-Zr both have a hexagonal close-packed structure with similar lattice parameters, their diffraction peaks may overlap in conventional XRD patterns. Therefore, the XRD results should be interpreted together with the lath-like or acicular morphologies shown in Figure 4 and Figure 5 [19,20,21,22].

The variation in diffraction peak intensity of the remelted samples may also be jointly affected by grain refinement, preferred orientation and the sampled volume during XRD measurements; thus, the phase fraction cannot be determined solely from changes in peak height. According to Bragg’s law, a shift in diffraction peaks toward higher angles corresponds to a decrease in interplanar spacing [23].

The vertical interface microstructure of 400 W laser remelting in air at different scales is shown in Figure 4. The maximum depth of laser remelting in the air is 407.08 μm. The microstructure of the remelting zone and the heat-affected zone can be shown in Figure 4b–f. As can be seen from Figure 4b–e, there are a large number of parallel or interleaved α plates and Widmanstätten structures in the laser remelting region.

Figure 5 shows the cross-sectional microstructure of the Zr60702 sample subjected to underwater laser remelting at 400 W. From Figure 5a in the low-magnification graph, three regions with different morphologies (shown in the white area) can be observed with a maximum remelting depth of 191.35 μm, which is only about one-third of the laser remelting depth in air. The microstructural details of the remelted zones as well as the heat-affected zone can be further revealed in the pictures in Figure 5b–f. The Zr60702 base metal consists of α-equiaxed grains with a tightly pushed hexagonal (HCP) structure. After laser remelting, the microstructure of the zirconium alloy sheet changes significantly.

Due to the different effects of laser energy in the aqueous environment, the peak temperatures in each region show different gradients. At a temperature higher than the melting point of Zr (1850 °C), the substrate is directly melted by laser irradiation, and α-Zr is replaced by β-Zr, as shown in Figure 5c,d. It can be seen that at this point it is a slat shape with an average size of 5 μm. Chai et al. [19] found that the β-Zr-to-α-Zr phase transition in Zr-4 was correlated with the cooling rate. It can be inferred that the phase transition from β-Zr to α-Zr occurs with the appearance of the Widmanstätten organization, and the morphology of the transformed Widmanstätten organization is related to the cooling rate. It has also been found that as the cooling rate varies, other different tissues appear, such as α’ martensite [20], Widmanstätten with a braided structure [21] and parallel-plate Widmanstätten [22]. The microstructure of the heat-affected zone is composed of small-sized irregularly shaped α’ tissue and a small amount of Widmanstätten α, as shown in Figure 5b–f.

It is necessary to distinguish between the temperature ranges for melting and solid-state phase transformation. During heating, the substrate first transforms from α-Zr to β-Zr after exceeding the α → β transition temperature, and melting occurs only when the temperature further exceeds the melting point. During cooling, the melt pool first solidifies into β-Zr, which subsequently transforms into α-Zr or supersaturated α′-Zr. Near the fusion line, the unmelted substrate can provide epitaxial nucleation sites, and microstructural growth is mainly governed by the large thermal gradient. In contrast, near the surface and the centre of the melt pool, the solidification rate and undercooling are higher, promoting more nucleation events and thereby producing finer lath-like/acicular structures with more dispersed orientations. The morphological gradient from the surface to the substrate essentially reflects the spatial variations in the thermal gradient G and interface growth rate R.

Compared with the air environment, the higher cooling rate underwater reduces the time available for solute diffusion in the β phase and for α-phase growth. As a result, the β → α transformation tends to proceed through diffusionless or short-range diffusion mechanisms, leading to a higher density of fine acicular α′ structures and refined Widmanstätten structures [19,20,21,22]. In air, the relatively slower cooling allows more time for α laths to grow and coalesce along similar orientations, making it easier to form coarser parallel lath bundles. Accordingly, a clear microstructural evolution sequence can be established: enhanced heat dissipation by water → shortened melt-pool lifetime and increased cooling rate → suppressed β-phase decomposition and lath refinement → increased microstructural interface density and dislocation density.

### 3.3. Hardness Measurements

**Figure 6:**
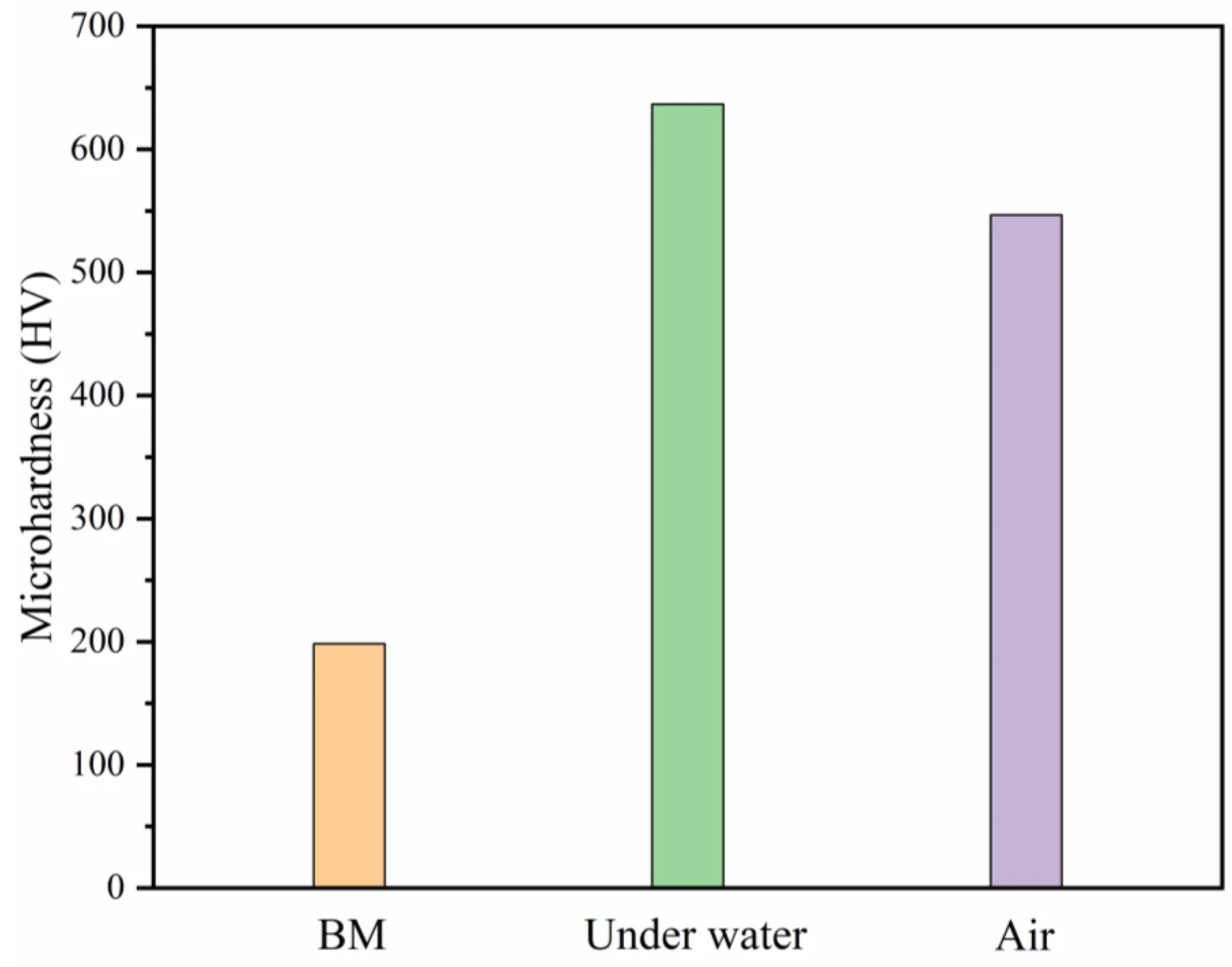
Microhardness of the base material and the samples remelted in air and underwater environments.

Figure 6 shows the hardness variation after laser remelting in air and underwater environments. The maximum hardness values of the underwater- and air-remelted samples were 636.7 ± 7.8 and 546.5 ± 12.3 HV, respectively, compared with 198.3 ± 6.7 HV for the base material (BM). These values were approximately 3.21 and 2.76 times that of the BM, respectively. Moreover, the maximum hardness of the underwater-remelted sample was approximately 1.17 times that of the air-remelted sample. The strengthening of the underwater-remelted layer cannot be attributed to a single factor. First, the fine acicular α′ structure and high-density lath interfaces shorten the mean free path of dislocations, thereby producing interface strengthening similar to the Hall–Petch effect. Second, the high dislocation density, lattice distortion and possible residual compressive stress induced by rapid phase transformation can increase the resistance to indentation deformation. Third, during underwater laser processing, if water decomposition and oxygen dissolution into high-temperature Zr occur, interstitial oxygen can produce pronounced solid-solution strengthening through its interaction with dislocations [23]. Differences in oxygen uptake between the air- and underwater-remelted samples may also influence their hardness because interstitial oxygen can strengthen α-Zr. However, EDX or XPS measurements were not conducted in this study. Therefore, the oxygen contents and their contribution to surface hardening could not be quantified, and oxygen-related strengthening is considered only a possible secondary mechanism.

### 3.4. Wear Resistance

**Figure 7:**
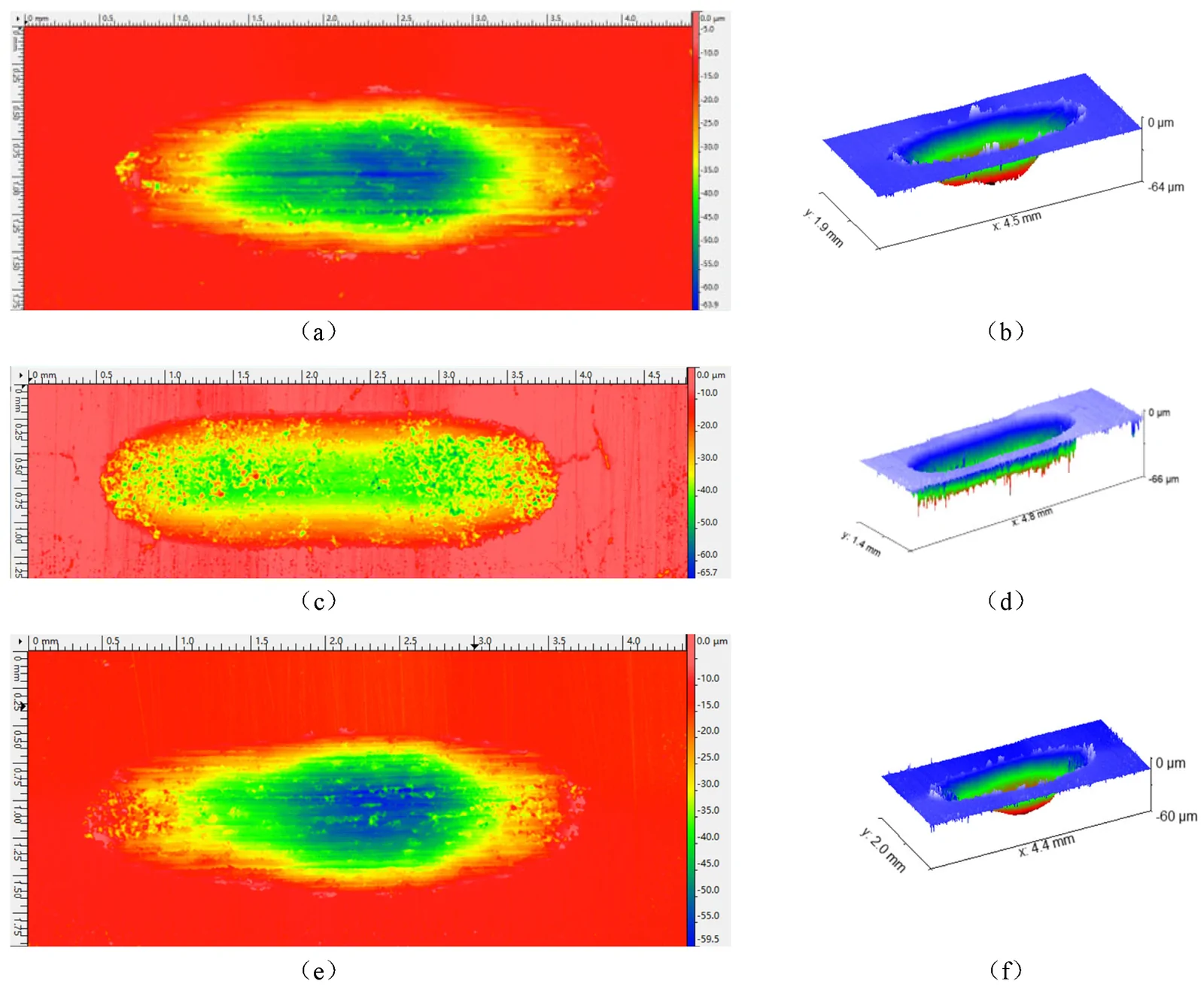
Two-dimensional surface maps and three-dimensional profiles of the wear tracks: (**a**,**b**) base material; (**c**,**d**) air-remelted sample; (**e**,**f**) underwater-remelted sample.

**Figure 8:**
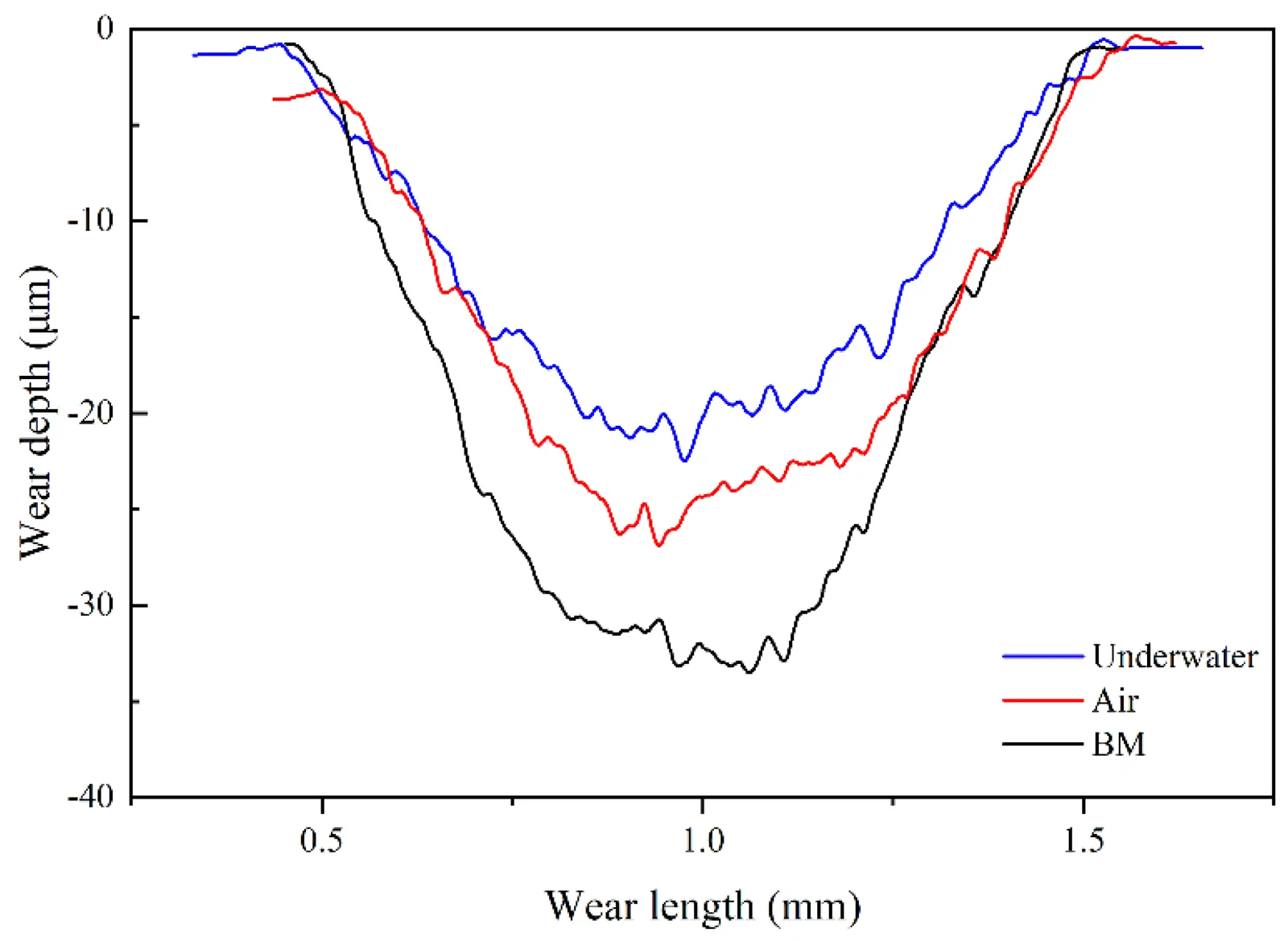
Two-dimensional profiles of the wear tracks of the base material and the samples remelted in air and underwater environments.

**Figure 9:**
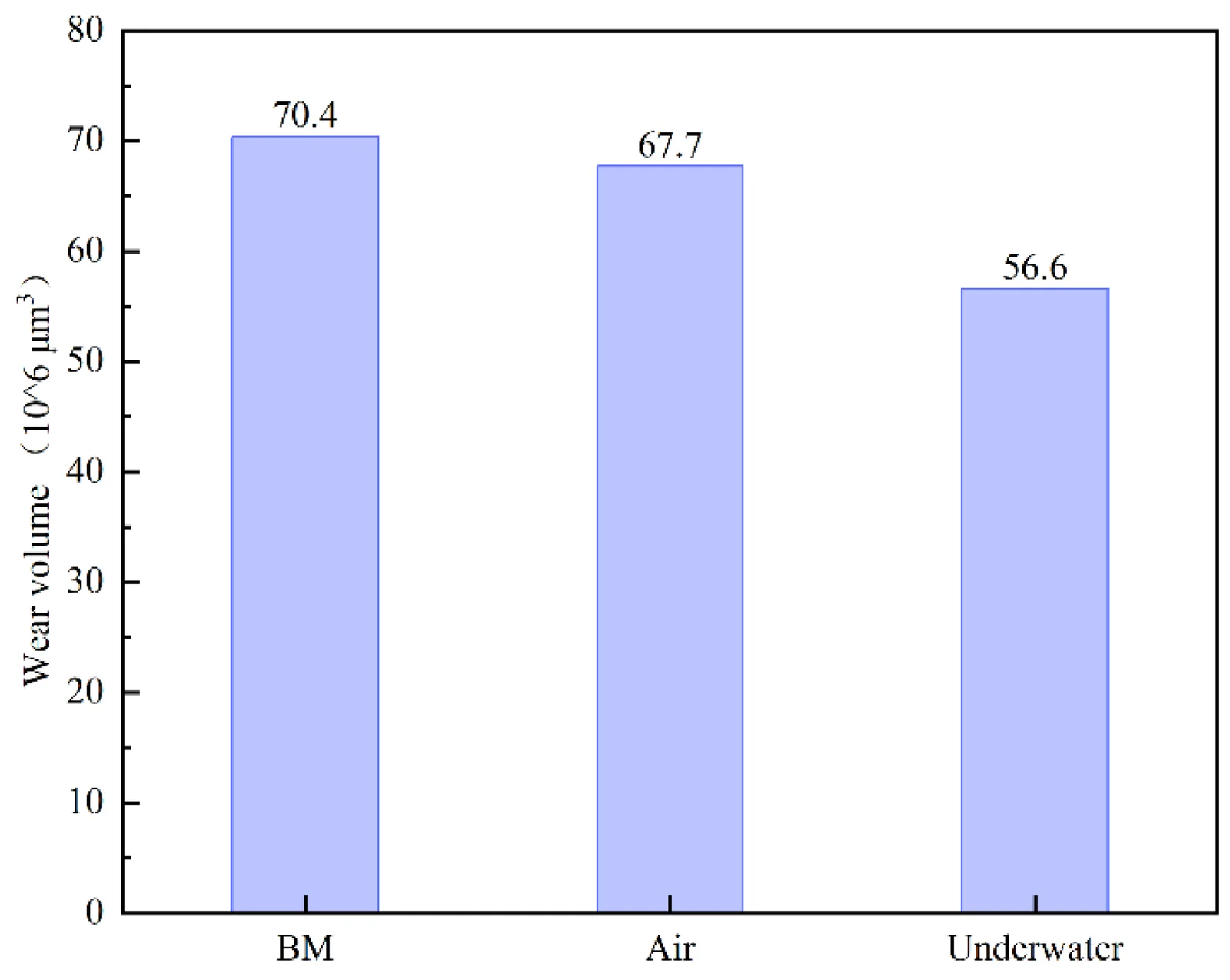
Comparison of the wear volumes of the base material and the samples remelted in air and underwater environments.

Zr60702 obtained a better hardening effect after laser remelting, which is conducive to improving wear resistance. Figure 7 shows the two-dimensional and three-dimensional wear curves of the zirconium alloy surface after multiple remeltings of the substrate with air and underwater. The wear profile of the substrate is shown in Figure 7a. The width and depth of the wear marks are 967 μm and 35.33 μm, respectively. The wear track of the air-remelted sample had a width of 1004 μm and a depth of 27.49 μm, as shown in Figure 7b. The width and depth of underwater laser remelting sample are 991 μm and 23.39 μm, respectively, as shown in Figure 7c. The two-dimensional interface of friction and wear depth is shown in Figure 8. Compared with the substrate, both air-remelted and underwater-remelted samples exhibited markedly reduced wear-track depths, with the underwater-remelted sample showing the shallowest wear track. As shown in Figure 9, the wear volumes of the substrate, air laser-remelted sample and underwater laser-remelted sample were 7.04 × 10^7^ μm^3^, 6.77 × 10^7^ μm^3^ and 5.66 × 10^7^ μm^3^, respectively. Compared with the substrate, the wear volume of the underwater laser-remelted sample was reduced by 19.6%. Under the same normal load and sliding distance, the Archard wear model indicates that the wear volume is approximately inversely proportional to material hardness [24,25]. After the hardness of the remelted layer increased, the penetration depth of the counterpart ball and the degree of surface plastic ploughing were reduced. In addition, fine lath interfaces and a high dislocation density could suppress subsurface shear deformation and the propagation of adhesive junctions. As a result, the underwater-remelted sample formed a shallower wear track, which is consistent with the wear-volume ranking shown in Figure 9.

However, the increase in hardness was not proportional to the reduction in wear volume: although the maximum hardness of the air-remelted sample was approximately 3.2 times that of the substrate, its wear volume decreased by only 3.8%. This indicates that the wear coefficient is not constant; the surface roughness after remelting, the stability of oxide/transfer films, local brittle spallation and the third-body effect of wear debris may all offset part of the hardness benefit [3,14,15]. The 19.6% reduction in wear volume of the underwater-remelted sample can reasonably be attributed to the synergistic effects of increased hardness, microstructural refinement and more uniform load bearing.

Adhesive wear and abrasive wear coexisted in all three samples, but the dominant mechanism and damage severity differed. The Zr60702 substrate had relatively low hardness, and the hexagonal close-packed α-Zr phase has a limited number of independently activatable slip systems at room temperature [26,27]. Therefore, local stress concentration and severe plastic deformation were likely to occur in the frictional contact region. Meanwhile, because zirconium is chemically active, adhesive junctions may form during contact with the GCr15 counterpart ball. With repeated formation and shearing of these junctions, the substrate underwent smearing, tearing and delamination. The detached particles then entered the contact interface and caused third-body ploughing. After air laser remelting, the Widmanstätten laths and transformation-induced defects increased the surface hardness, thereby reducing the penetration depth of the counterpart ball and the degree of macroscopic ploughing. However, the lath bundles formed in air were relatively coarse and showed distinct orientation partitioning. The plastic compatibility between different lath bundles was therefore limited, and under cyclic tangential stress, lath-bundle boundaries may act as preferential sites for microcrack initiation and local spallation. The hard debris generated by spallation can enhance three-body abrasive wear. Therefore, although the maximum hardness reached approximately 3.2 times that of the substrate, the wear volume decreased by only 3.8%, indicating that part of the hardness benefit was offset by local spallation and surface topographical fluctuations. In contrast, the fine acicular α′ structure formed by rapid underwater cooling possessed a higher density of lath interfaces and dislocations. This could markedly shorten the dislocation glide distance, restrict the extension of the friction-induced plastic zone into the subsurface, and disperse contact stress through fine laths with more distributed orientations, thereby suppressing adhesive-junction propagation, plastic ploughing and subsurface shear-induced spallation. If stable oxides were present on the remelted surface or a relatively compact transfer film formed during sliding, direct metal-to-metal contact and the tendency for adhesion could be further reduced.

## 4. Conclusions

In order to improve the surface hardness and wear resistance of the Zr60702 alloy, laser remelting in a water environment is proposed in this paper. By analyzing the hardness and wear resistance of the substrate, air and water, the following conclusions are drawn:

(1) Due to the refraction of the laser beam in the water environment and the accelerated heat dissipation along the water-layer direction, the underwater laser remelting depth and heat-affected zone width are significantly smaller than that in the air environment.

(2) The rapid cooling of the substrate in the laser remelting process was caused by accelerated heat dissipation in the water environment. The maximum hardness of the underwater-remelted sample reached 636.7 ± 7.8 HV, which was approximately 3.21 times that of the base material and 1.17 times that of the air-remelted sample.

(3) The wear volumes of the BM, air-remelted sample, and underwater-remelted sample were 7.04 × 10^7^μm^3^, 6.77 × 10^7^, and 5.66 × 10^7^ μm^3^, respectively. The wear volume of the underwater-remelted sample was reduced by 19.6% compared with that of the BM. This improvement was attributed to the accelerated cooling underwater, which promoted the formation of finer acicular α′-Zr and refined Widmanstätten structures. The resulting increase in hardness and load-bearing capacity suppressed subsurface plastic deformation and ploughing during sliding.

## Figures and Tables

**Table 1:** Nominal chemical composition of commercially pure Zr60702 according to the manufacturer’s material certificate (wt.%).

Elements	Zr + Hf	Hf	Fe + Cr	O	H	N	C
Contents	≥99.01	≤4.5	≤0.20	≤0.016	≤0.005	≤0.025	≤0.05

## Data Availability

The data presented in this study are available on request from the corresponding author. The data are not publicly available due to privacy.
